# Mechanosensitive recruitment of stator units promotes binding of the response regulator CheY-P to the flagellar motor

**DOI:** 10.1038/s41467-021-25774-2

**Published:** 2021-09-14

**Authors:** Jyot D. Antani, Rachit Gupta, Annie H. Lee, Kathy Y. Rhee, Michael D. Manson, Pushkar P. Lele

**Affiliations:** 1 Artie McFerrin Department of Chemical Engineering, Texas A&M University, College Station, TX 77843-3122 USA; 2 Department of Biology, Texas A&M University, College Station, TX 77843-3258 USA; 3 Present Address: Department of Ecology & Evolutionary Biology, Yale University, New Haven, CT 06520-8106 USA; 4 Present Address: Department of Molecular, Cellular, and Developmental Biology, Yale University, New Haven, CT 06520-8103 USA

**Keywords:** Motor protein function, Chemotaxis, Bacterial physiology, Cellular microbiology

## Abstract

Reversible switching of the bacterial flagellar motor between clockwise (CW) and counterclockwise (CCW) rotation is necessary for chemotaxis, which enables cells to swim towards favorable chemical habitats. Increase in the viscous resistance to the rotation of the motor (mechanical load) inhibits switching. However, cells must maintain homeostasis in switching to navigate within environments of different viscosities. The mechanism by which the cell maintains optimal chemotactic function under varying loads is not understood. Here, we show that the flagellar motor allosterically controls the binding affinity of the chemotaxis response regulator, CheY-P, to the flagellar switch complex by modulating the mechanical forces acting on the rotor. Mechanosensitive CheY-P binding compensates for the load-induced loss of switching by precisely adapting the switch response to a mechanical stimulus. The interplay between mechanical forces and CheY-P binding tunes the chemotactic function to match the load. This adaptive response of the chemotaxis output to mechanical stimuli resembles the proprioceptive feedback in the neuromuscular systems of insects and vertebrates.

## Abstract (web summary)

It is unclear how bacterial cells adapt the reversible switching of flagellar motor rotation to environments of different viscosities. Here, Antani et al. show that flagellar mechanosensors allosterically control the motor’s binding affinity for the chemotaxis response regulator, CheY-P, to adapt flagellar switching over varying viscous loads.

## Introduction

*E. coli* swim by rotating helical flagellar filaments with transmembrane flagellar motors. The flagellar motor consists of a stator and a rotor. The stator is made up of several independent stator units, with each unit capable of rotating the motor. The rotor is a multimeric complex of several different types of proteins^1^. The chemotaxis response regulator, CheY-P, binds cooperatively to the cytoplasmic interface (C-ring) of the motor to increase the probability of clockwise (CW) rotation in an otherwise counterclockwise (CCW) rotating motor^2^. This switch output is called the CW_bias_. Modulation of the CW_bias_ is the basis for chemotaxis—the migration toward favorable chemical environments. The chemotaxis network modulates the CW_bias_ in response to chemical stimuli by controlling the level of CheY-P.

The basal CW_bias_ must be maintained within its dynamic range (0 < CW_bias_ < 1) for chemotaxis: cells of *E. coli* in which the motors remain locked in CCW (CW_bias_ = 0) or CW (CW_bias_ = 1) rotation fail to respond to chemical stimuli^3^. We previously showed that a sudden increase in the viscous load on the motor inhibits switching, causing it to rotate exclusively CCW^4^. Within a few minutes, the motor recruits additional stator units, which increase the torque delivered to the motor. During this time, the switch adapts to the original steady-state CW_bias_ without a change in the level of intracellular CheY-P.

Here, we investigated whether mechanosensitive stator recruitment influences the interactions of CheY-P with the C-ring, a complex structure that contains three proteins—FliG, FliM, and FliN^5^. We discovered that more CheY-P binds to the C-ring when more stator units bind to the motor in response to increased torque. However, neither recruitment of more FliM, as has been seen in response to chemotaxis signals^6^, nor changes in the proton flux that powers motor rotation, is responsible for the changes in CheY-P binding affinity. Instead, our data suggest that increased mechanical stress generated by the stator units on the FliG_C_ domain regulates CheY-P binding to FliM_N_/FliN. This mechanism enables the cell to adapt its basal CW_bias_ precisely, which helps fine-tune the motor sensitivity such that the cell is able to respond to chemical stimuli over a range of viscous loads. We suggest that this process is functionally analogous to, although mechanistically completely distinct from, the proprioceptive feedback that controls the neuromuscular circuitry in animals.

## Results

### Mechanosensitive stator recruitment modulates CheY-P binding

**Fig. 1:**
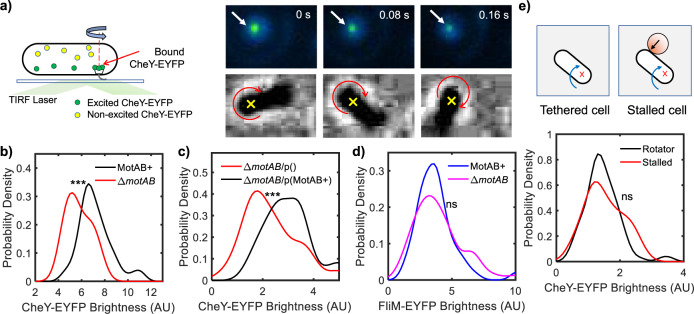
Binding of CheY-P to the motor increases with the number of bound stator units. **a** We monitored the rotation of tethered cells simultaneously in the TIRF and phase channels. The fluorescent moieties (CheY-EYFP) were visible in the evanescent field as they bound to the motor: The top row shows a time series of TIRF images for a single tethered cell. The arrow points to CheY-P bound to the motor. The corresponding phase contrast images are shown in the bottom row. The ⨯ shows the point of tethering around which the cell rotates. **b** The intensity of CheY-P localized around each motor was quantitatively estimated from the TIRF images (see *Methods*). The probability-density estimates are indicated for a strain carrying functional stators (MotAB+ strain, black curve, *n* = 50 independent motors) and a strain lacking stator proteins (Δ*motAB* strain, red curve, *n* = 48 independent motors). The difference in the mean intensities in the two strains was significant (***p-value = 1.3 × 10^−7^, <0.001). The mean number of CheY-P molecules bound to the motor in the absence of stator units was lower than that in the presence of stators units. **c** We compared CheY-EYFP binding to the motors in the Δ*motAB* strain transformed with a plasmid encoding MotA and MotB with that in a Δ*motAB* strain transformed with an empty plasmid. The strain lacking MotA-MotB (red curve, *n* = 108 independent motors) had significantly lower intensities (***p-value = 1.7 × 10^−5^, <0.001) than the strain expressing MotA-MotB (black curve, *n* = 46 independent motors). **d** We measured the localization of FliM-EYFP-FliM in each tethered cell. The difference in the mean intensities in the motors of a strain carrying functional stators (blue curve, *n* = 75 independent motors) and a strain lacking stator proteins (magenta curve, *n* = 115 independent motors) was not statistically significant (ns, *p*-value > 0.05). **e** Tethered cells were stalled by obstructing the rotation with optically trapped beads. The difference in the mean CheY-EYFP intensities in rotating cells (black curve, *n* = 86 independent motors) and stalled cells (red curve, *n* = 36 independent motors) was not statistically significant (ns, *p*-value > 0.05). Source data are provided as a Source Data file.

We employed the tethered cell assay to determine whether mechanosensitive stator recruitment modulates the interactions of CheY-P with the C-ring (Fig. 1a). We worked with a Δ*cheRcheBcheYcheZ* strain, called the MotAB+ strain hereafter, and transformed it with a pTrc99A vector encoding *cheY* fused with *eyfp* (see “Methods” section). The CheY-EYFP fusion is functional and induces switching in the motors in the absence of native CheY^7^. The viscous load on the motor in a tethered cell is significant (rotational drag coefficient ~150 pN nm s), and the high resistance to rotation causes the motor to recruit a full complement of ~11 stator units in ~5 min^8^. Hence, we waited 10 min after tethering to allow stator recruitment to complete. Next, cells were excited with a 514 nm laser in the TIRF mode to visualize CheY-EYFP localization around each tethered motor, and the fluorescence signals were recorded with an EMCCD camera (Fig. 1a). Simultaneous recording of cell rotation in the phase-contrast channel helped locate precisely the tether around which the cell body rotated. Mapping the spatial coordinates in the phase-contrast images onto the TIRF images allowed us to locate each motor accurately in the TIRF channel. As we recorded a time-series in the TIRF channel, cell rotation was also visible in the TIRF images. This enabled us to confirm the motor location independently in the TIRF channel (see “Methods” section).

We employed previously developed MATLAB routines to quantify the intensity of the fluorescent punctum in each tethered cell^9^. Briefly, we corrected for the background fluorescence and determined the total pixel intensities within a 350 nm mask placed around each motor (see “Methods” section). The total intensity of each punctum at the point of the tether indicated the total number of fluorophores bound to the motor^9^. The mask ensured that we did not include neighboring fluorescent moieties when calculating the intensities. The probability-density estimates for the intensities obtained from *n* = 50 motors are indicated in Fig. 1b.

Each stator unit is made up of five MotA subunits and two MotB subunits^10,11^. A stator-less motor was generated by deleting *motAmotB* from the MotAB+ strain. We visualized CheY-EYFP localization in tethered cells of this strain to determine how the absence of stator units affects CheY-P interactions with the motors. In the absence of stators, tethered cells diffuse rotationally. To identify the point of tether, we forced the cells to rotate with hydrodynamic flows and recorded their movements in the phase channel. We did this by turning the flow of motility buffer into the perfusion chamber on and off several times. Each time, the non-motile cells that were tethered by their flagella rotated to align with the direction of the flow. Those that were stuck to the surface did not move. With this approach, we could reliably distinguish between the cells that were tethered by their flagellar stubs and those that merely adhered to the surface. We recorded the forced rotation of the cell, which allowed the point of tether to be quantified from the phase-contrast data for each cell. The probability-density estimates of CheY-EYFP localization at the motors in the Δ*motAmotB* strain are shown in Fig. 1b. As can be seen, more CheY-EYFP molecules bound to motors that rotated with a full complement of stator units relative to the motors of non-motile cells that lacked stators.

Next, we determined whether the differences observed in CheY-P binding in the two strains seen in Fig. 1b could be attributed to differences in the phosphorylation levels of CheY, to differences in the activity of the chemotaxis kinase CheA, or to some other unknown effects. We transformed the Δ*motAmotB* strain with a pBAD34 vector carrying *motAmotB* transcribed from the *araBAD* promoter to make expression of MotA and MotB inducible with arabinose^12^. This plasmid is compatible with the pTrc99A vector. This yielded the Δ*motAmotB*/p(MotAB+) strain (see “Methods” section). A control strain was prepared by transforming the Δ*motAmotB* strain with an empty pBAD34 plasmid vector, which yielded the Δ*motAmotB*/p() strain. We repeated our measurements of CheY-P binding to motors in these two strains, using the protocols described previously. As seen in Fig. 1c, CheY-P molecules bound to the motor in greater numbers when the stator proteins were expressed than when they were absent. We conclude that the remodeling of the stator promotes CheY-P binding to the motor.

### The FliM ring does not remodel when the stator remodels

The FliM complex is capable of remodeling by recruiting FliM subunits to adapt to variations in CheY-P levels caused by strong chemoattractants^6,9^. We thus asked whether mechanosensitive stator recruitment causes recruitment of additional FliM, thereby increasing CheY-P binding. We performed TIRF measurements to compare the number of FliM subunits in individual motors in the presence and absence of the stator units. We employed a *ΔcheY* strain that carries a *fliM-eyfp-fliM* allele in place of the chromosomal *fliM* gene (see “Methods” section). The *fliM-eyfp-fliM* gene contains an internal fusion to *eyfp* that does not interfere with motor rotation^13^. We compared the FliM-EYFP-FliM fluorescence intensities in tethered cells of this strain and in cells of a strain deleted for *motAmotB*, using the protocols discussed in previous sections. The absence of CheY locked the rotation of the motors in the CCW-only direction, ensuring that changes in the direction of rotation did not contribute to FliM remodeling^9^. As shown in Fig. 1d, the difference in the mean fluorescence intensities in the two strains was not significant (*p* > 0.05). Thus, the number of FliM subunits in the motor did not change with the number of bound stator units. The similarity in the intensities in the presence and the absence of the stator also ruled out the possibility that the fluorescence intensity of individual FliM-EYFP-FliM molecules was somehow different in the presence and absence of MotA-MotB. These data suggest that the affinity of CheY-P for FliM increases with an increasing number of stator units bound to the motor.

### CheY-P binding does not depend on proton flux or motor rotation

Flagellar rotation is powered by the proton motive force—the flux of protons through the stator from the periplasm into the cytoplasm yields the free energy required to rotate the motor^14^. Stator recruitment increases the flux of protons. We explored whether this increased proton flux influences the affinity of CheY-P for FliM. To do this, we compared CheY-P binding in rotating and stalled motors. Stalled motors retain a full complement of bound stator units just like rotating tethered cells, as the load is similar^15^. However, the proton flux is expected to decrease sharply when rotation is prevented.

We tethered cells of the MotAB+ strain as before and provided adequate time for stator remodeling. Next, we optically trapped large latex beads (4.5 μm diameter) and placed them in the path of a rotating cell to obstruct its rotation (Supplementary Movie 1). After 2 min, we imaged CheY-EYFP localized at the motors in the stalled cells and quantified the fluorescence intensities. We compared these intensities with those observed at the motors in tethered cells in the vicinity that rotated freely. There was no significant difference in the mean CheY-EYFP fluorescence intensities in the stalled and rotating motors (Fig. 1e). This result suggests that the reduced affinity of CheY-P for FliM in the absence of stators was not due to the lack of proton flux or motor rotation. Instead, it is likely that mechanical force regulates CheY-P binding as torque on FliG increases with an increasing number of bound stator units, when the motor rotates under a high viscous load^16^.

### Mechanosensitive stator recruitment promotes CW rotation

To determine the functional consequences of the mechanosensitive binding of CheY-P, we measured the CW_bias_ in cells when the torque was differentially controlled by varying the number of stator units bound to the motor (*N*_st_). We employed a Δ*motA-motB* strain of *E. coli* transformed with the pBAD34*-motAmotB* plasmid. The strain also lacks chromosomal alleles for the methyltransferase (CheR), methylesterase (CheB), and the phosphatase (CheZ), which ensures a high level of phosphorylated CheY in the cell. It carries the *cheY* allele on the chromosome. Using this strain allowed us to measure variations in the CW_bias_ over its full dynamic range (0–1). We attached 2 μm beads to individual flagella and waited an adequate time (~10 min) for mechanosensitive stator recruitment to complete (see “Methods” section). At steady state under a high viscous load (for example, a 2 μm bead or a tethered cell), the motor contains ~8–11 stator units in a wild-type cell^16^. However, by carefully limiting the production of MotA-MotB proteins, we could generate motors containing anywhere from 1 to 11 stator units.

**Fig. 2:**
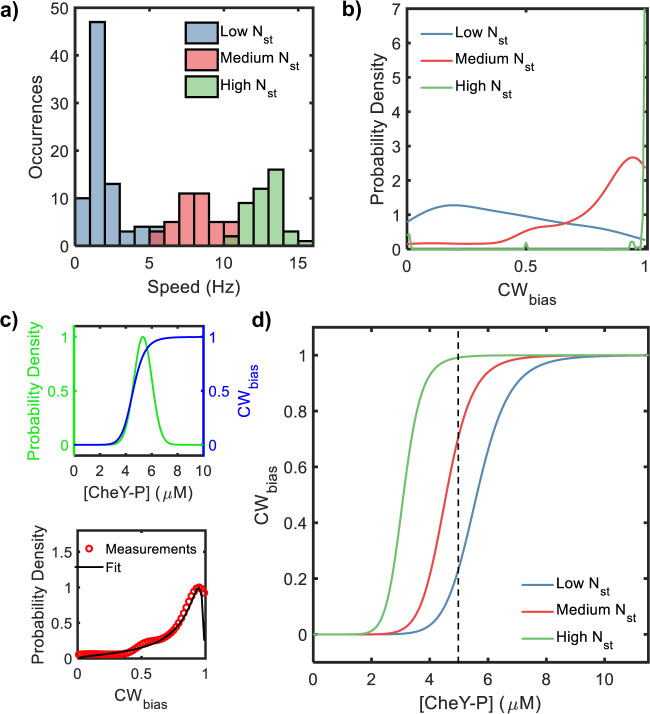
Motor response curves shift with the number of bound stator units. **a** The number of stator units bound to individual motors was varied by controlling the expression of MotA-MotB from an inducible plasmid-borne promoter (see “Methods” section). We probed motor rotation with 2 μm beads (high viscous load). Data on a total of *n* = 165 independent motors were collected and binned into three groups as per the speed of rotation, as indicated by the different colors: low speeds (*n* = 81 independent motors, average torque ~ 270 pN nm, blue data), medium speeds (*n* = 40 independent motors, average torque ~ 1150 pN nm, red data), and high speeds (*n* = 44 independent motors, average torque ~ 1800 pN nm, green data). Each stator unit adds ~1.1–1.3 Hz to the overall rotation speed. **b** The probability density for the CW_bias_ is indicated for each speed group. The distribution shifted to increased CW_bias_ with an increasing number of bound stator units (*N*_st_). **c** *Top*: The variation in the CheY-P levels in a population (green curve) was described by a normal distribution (*μ* = 5.3 ± 0.5 μM, mean ± SEM; *σ*/*μ* = 0.13). The CW_bias_ versus CheY-P relationship is described by a Hill equation (blue curve) parameterized by the Hill coefficient $h$ and the dissociation constant ${K}_{{{{{\rm{{D}}}}}}}$. Together, these relationships provide an equation for the distribution of CW_bias_ (see Supplementary Note 1). *Bottom*: The value of ${K}_{{{{{{\rm{D}}}}}}}$ for each of the three *N*_st_ groups was calculated from a least-square fit of the analytical CW_bias_ distribution to the corresponding experimental data. The plot shows a representative fit (black curve) to the experimental data (symbols) for the medium value of *N*_st_. We assumed a constant $h$ = 10^17^. **d** The CW_bias_ versus CheY-P curves calculated from the fitted ${K}_{{{{{{\rm{D}}}}}}}$ values are shown. The curves shift left with increasing *N*_st_ (the number of stator units bound to the motor). At a representative [CheY-P] = 5 μM (dashed black line), the CW_bias_ = 0.24, 0.71, and 0.99 for low, medium, and high *N*_st_, respectively. Source data are provided as a Source Data file.

We measured the rotational speeds of a total of *n* = 165 motors over the different induction levels, as described in “Methods” section. We estimated the *N*_st_ for each motor by dividing the average speed by 1.3 Hz, which is the approximate increment in the rotational speed when a single stator unit is added to the stator^16^. We binned the data into three groups: a low *N*_st_ group consisting of 1–4 stator units, a medium *N*_st_ group consisting of 5–8 units, and a high *N*_st_ group (>8 units). The distributions of speeds for the three groups are indicated in Fig. 2a. The CheY-P levels vary significantly among Δ*cheRcheBcheZ* cells because of intrinsic random differences in the expression of the genomic *cheY* gene from cell to cell. Thus, the CW_bias_ values are widely distributed over a population of cells^17^. We measured the distributions of the steady-state CW_bias_ in each group. Figure 2b shows the kernel-density estimates for the CW_bias_ distributions; the raw data are available in the Supplementary text (Supplementary Fig. 1). The CW_bias_ was distributed predominantly at ~1 at high *N*_st_ (>8 stator units, average torque ~ 1800 pN nm). At a medium *N*_st_ (5–8 stator units, average torque ~ 1150 pN nm), the average bias was lower (0.8 ± 0.04, mean ± SEM). At the lowest *N*_st_ (1–4 stator units, mean torque ~ 270 pN nm), the average CW_bias_ dropped to 0.4 ± 0.04 (mean ± SEM). Thus, variations in the motor torque modified the distributions of the CW_bias_ in populations of cells.

### Mechanosensitive stator recruitment tunes motor sensitivity

Next, we quantified how torque modulates the dependence of the CW_bias_ on CheY-P levels from the distributions of the CW_bias_ in Fig. 2b. The CW_bias_ vs. [CheY-P] relationship is well described by the Hill equation^17,18^. As the intracellular CheY-P levels are independent of the number of stator units, the variations in the CW_bias_ distributions for the different torque values (Fig. 2b) arise entirely due to changes in the CW_bias_ vs. CheY-P relationship with the number of stator units bound to the motor.

To determine how the CW_bias_ vs. CheY-P relationship depends on torque, we derived an analytical expression for the distribution of the CW_bias_ in a population of cells, following our previous work^17^. To do this, we assumed a normal distribution of CheY-P with a mean $\mu$ and a spread of $\sigma$. We combined the distribution with the Hill equation that describes the CW_bias_ versus CheY-P relationship (Fig. 2c) to yield an analytical form for the CW_bias_ distribution in a population of cells (see Eqs. 1 and 2 in Supplementary Note 1). We first fitted Eq. 2 to the CW_bias_ distribution for the high torque (high *N*_st_) group in Fig. 2b, with $\mu$ as a free parameter. We assumed *σ* = 0.13 *μ*, a Hill coefficient value ($h$) = 10, and a dissociation constant value (${K}_{{{{{{\rm{D}}}}}}}$) = 3.1 μM based on previous studies^17,18^. A nonlinear least-square fit yielded $\mu$ = 5.3 ± 0.5 μM (mean ± SEM) for the *ΔcheRcheBcheZ* strain, which is reasonable given the higher levels of CheY-P phosphorylation in this strain compared to the wild type. Next, we assumed the same values of $\mu$ and $\sigma$ for the remaining *N*_st_ groups, as the distribution of [CheY-P] is not expected to depend on the number of stator units. Nonlinear least-square fitting with ${K}_{{{{{{\rm{D}}}}}}}$ as a free parameter yielded ${K}_{{{{{{\rm{D}}}}}}}$ = 4.5 ± 0.0 for medium torque (medium *N*_st_) and = 5.6 ± 0.0 for low torque (low *N*_st_), see Supplementary Table 1. The dissociation constant ${K}_{{{{{{\rm{D}}}}}}}\,$for the CheY-P/motor interaction thus decreased with increasing torque.

Next, we analytically calculated the CW_bias_ values over varying CheY-P levels for each *N*_st_ group by plugging in the corresponding fitted ${K}_{{{{{{\rm{D}}}}}}}$ value into the Hill equation (Eq. (1) and Supplementary Note 1). The three CW_bias_ versus CheY-P curves are shown in Fig. 2d. The curves shift leftward as the motor recruits more stator units following an increase in load. The high *N*_st_ case (green curve) correctly represents the CW-only rotation of motors with a full complement of stator units (Fig. 2a). The ${K}_{{{{{{\rm{D}}}}}}}$ is highest for the case of lowest torque (~270 pN nm). The shift in motor response curves with increasing torque indicates that the CW_bias_ increases as more stator units are recruited.

## Discussion

In our previous work, we observed that the immediate effect of increasing the load on a flagellar motor is to slow its rotation and to decrease its CW_bias_. The motor adapts to the higher load by recruiting additional MotA-MotB stator units, which increases the speed of rotation and the CW_bias_ to steady-state values^4^. Here, we showed that the motor adapts the CW_bias_ by increasing its affinity for the chemotaxis response regulator CheY-P, whose binding to the motor promotes CW rotation.

Our measurements suggest that there is no significant difference in CheY-P binding to motors rotating under high torque relative to motors stalled with optical traps (Fig. 1e). Although it is possible that some protons leak through the stator in a stalled motor, the high duty ratio under high viscous loads ensures that the proton flux through a stalled motor is very low. The recent model for stator function by rotation of the MotA pentamer of a stator unit around a centrally located MotB dimer, which is held stationary by attachment to the cell wall, also predicts that stator units in a stalled motor will not conduct ions^10,11,19^. Thus, our data suggest that it is not the increased proton flux caused by the mechanosensitive recruitment of stator units that influences CheY-P binding. Instead, it is likely the torque (force) delivered by the stator to the rotor that modulates CheY-P binding.

**Fig. 3:**
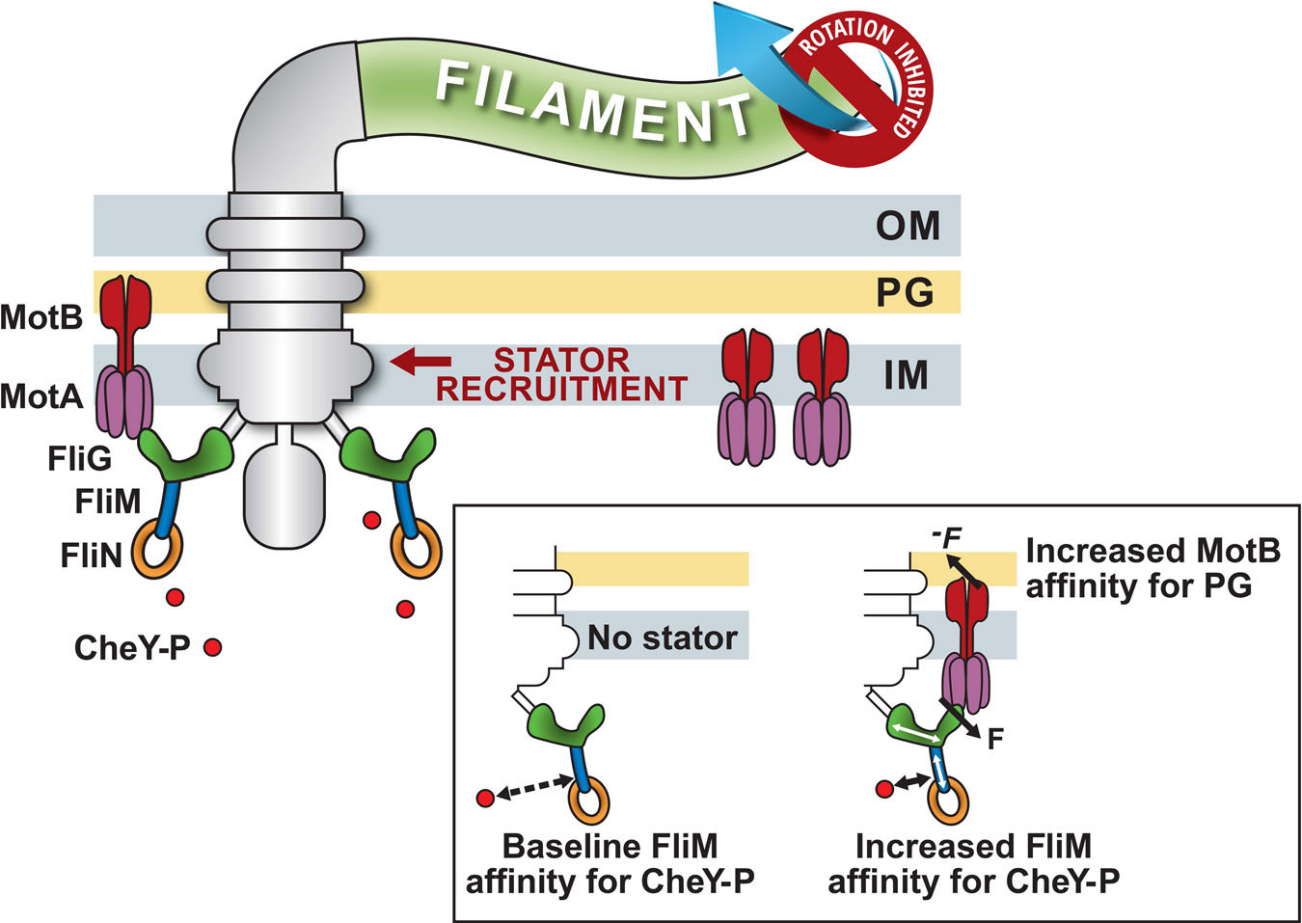
Model for mechanosensitive control in the flagellar motor. Independent stator units (MotA purple, MotB maroon units) deliver torque by interacting with the C-terminal domain of FliG (green) to rotate the transmembrane flagellar motor. The conformational state of the FliG ring determines the direction of rotation. The default conformation is CCW; binding of CheY-P to the FliM (blue) and FliN (orange) complexes increases the probability that the FliG ring will adopt a CW conformation. An increase in the viscous resistance to the rotation of the extracellular flagellar filament causes the transmembrane flagellar motor to recruit additional stator units. Inset: *Left*: In the absence of stator units, the affinity CheY-P binding is at a baseline level. *Right*: Increased mechanical force (*F*) on the FliG_C_ domain due to the mechanosensitive recruitment of a stator unit induces conformational shifts (white arrows) in FliM/FliN, increasing their affinity for CheY-P. According to our model, the equal and opposite force (−*F*) strengthens the association of the peptidoglycan-binding domain of MotB with the cell wall, increasing the dwell time of the stator unit at the motor^8^. The torque-mediated increase in CheY-P affinity compensates for the increased resistance to changes in the conformation of the FliG ring, thereby maintaining a constant motor sensitivity (basal CW_bias_).

Each stator unit increases the total force it delivers to a FliG subunit from ~0.5 pN at very low loads to ~10 pN at high viscous loads^4^. In a stator with a full complement of 11 stator units, this corresponds to an increase of ~110 pN over the FliG ring. We previously proposed that increased torque delivered by each stator unit following the adhesion of the flagellum to a surface is the basis for mechanosensitive stator recruitment^8,20^. As the force delivered by MotA to FliG causes an equal and opposite force, where MotB anchors to the cell wall, the increased torque strengthens its interactions with the peptidoglycan-binding domain of MotB (Fig. 3). There is experimental support for this notion^8,21–23^.

Here, we propose that the force felt by the C-terminal domain of a FliG subunit is transmitted to the FliM subunit. These conformational shifts increase the affinity of the N segment of FliM, which lies ~15 nm away from the C-terminal domain of FliG, for CheY-P (Fig. 3). When the force is weaker, as at very low viscous loads^24^, or when no stator units are present, these allosteric conformational changes do not occur. The affinity for CheY-P increases when the torque increases: ${K}_{{{{{{\rm{D}}}}}}}$ decreases by ~2.5 μM with an increase in torque of ~1530 pN nm (Fig. 2d). This increased affinity for CheY-P, coupled with the cooperativity in CheY-P binding and the ultrasensitive motor response curve, causes a major change in CW_bias_ (Fig. 2d).

**Fig. 4:**
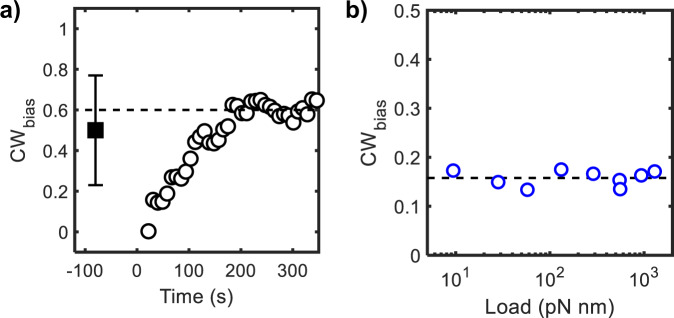
Precise adaptation to mechanical stimuli. **a** The CW_bias_ at low viscous load was measured to be 0.5 ± 0.27 (mean ± SD, solid square, *n* = 41 independent motors) in a *ΔcheRcheB* strain by monitoring the rotation of 300 nm beads. The standard deviation is indicated. The post-stimulus data (open circles, *n* = 9 independent motors) from ref. ^4^ shows the adaptation in CW_bias_ when the load is suddenly increased by tethering a cell (at *t* = 0 s). **b** The steady-state CW_bias_ for wild-type cells (open circles), calculated from the transition rates between CW-CCW directions of rotation^26,27^, is indicated as a function of load. Dotted line is a guide to the eye. Source data are provided as a Source Data file.

The mechanosensitive binding of CheY-P explains our previous observations of a sharp decrease and subsequent adaptation in the CW_bias_ when a motor that was initially rotated by a single stator unit is mechanically stimulated^4^. The CW_bias_ adaptation in a Δ*cheRcheB* strain is reproduced in Fig. 4a, where the viscous load was changed at *t* = 0 s. The time required for adaptation is similar to the time it takes the stator to fully remodel. To determine the pre-stimulus CW_bias_, we measured the average CW_bias_ at low loads by measuring the rotation of 300 nm beads in the Δ*cheRcheB* strain. As is evident in Fig. 4a (*t* < 0 s), the pre-stimulus and post-stimulus CW_bias_ is similar. This suggests that the adaptation in CW_bias_ to the increase in viscous load is precise and independent of CheR and CheB. We propose that, upon an increase in the viscous load, the increased torque delivered by the single stator unit physically obstructs the FliG subunit from undergoing a change to the CW conformation. This idea is consistent with a recent model that assumes that torque increases the difference in free energy (ΔG_CCW-CW_) between the lower-energy CCW conformation and the higher-energy CW conformation of the FliG ring^25^. Our results further indicate that the *K*_D_ for CheY-P binding decreases as the torque increases during stator recruitment. The torque-dependent increase in the affinity of FliM for CheY-P compensates for the elevated ΔG_CCW-CW_, leading to precise adaptation in the CW_bias_ to mechanical stimuli.

The data in Fig. 4a suggest that the mechanosensitive binding of CheY-P could compensate for any change in load to maintain a constant basal CW_bias_. To test this, we calculated the CW_bias_ for wild-type cells from previous studies that reported the transition frequencies for CW-to-CCW and CCW-to-CW reversals over a wide range of loads (see Supplementary Note 2 and^26,27^). Our calculations indicate that the CW_bias_ in the wild type was indeed independent of the viscous load (Fig. 4b). Thus, the torque-mediated interplay between stator recruitment and mechanosensitive CheY-P binding ensures that the CW_bias_ of the motor remains within the dynamic range of the response (0 < CW_bias_ < 1) even when the viscous load changes. This adaptation facilitates chemotaxis over a wide range of viscosity. In bacteria in which the flagellar motor contains a constant numbers of stator units, such as *Helicobacter pylori*^28^, load-dependent stresses may still tune the affinity of FliM for CheY-P. Future research will be required to test this possibility.

In addition to chemotaxis, an intermediate value of the CW_bias_ (0 < CW_bias_ < 1) is necessary for inducing bacterial swarming^29^, a surface-associated form of group motility^30^. The mechanosensitive binding of CheY-P to FliM likely maintains homeostasis in switching under the increased viscous drag near a surface, thereby aiding in the transition from a planktonic state to the surface-associated swarmer state. Mechanosensitive CheY-P binding may also prevent swimming cells from becoming hydrodynamically trapped at surfaces, as exclusively smooth-swimming cells are^31^, by inducing tumbles.

There are several similarities between the phenomena discussed in this work and proprioception in neuromuscular systems, which enables an organism to detect its velocity and position in space^32,33^. For example, proprioceptive feedback in the motor neurons that enervate the leg muscles in an insect maintains maximal sensitivity to different mechanical loads, allowing the insect to maintain its posture and grip when walking on the floor or the ceiling^34^. Likewise, resetting of the flagellar motor’s CW_bias_ maintains maximal sensitivity of the chemotaxis network to different viscous loads. This allows the cell to adapt to changes in its swimming speeds, for example, when entering the highly viscous mucous layer coating the intestine. Similar to proprioreceptors, the flagellar motor encodes the resistance to its motion as load and relays information regarding changes in the cell’s position in space, for example, when the flagellum adheres to a surface^20,35^. Thus, proprioception is likely critical for *E. coli* in chemotaxis and in surface sensing.

## Methods

### Strains and plasmids

All strains are derivatives of *E. coli* RP437. They carry a sticky variant (*fliC*^*st*^) of the flagellin gene, which allows cells to be tethered to glass surfaces and to latex beads via their flagellar filaments. Chromosomal modifications were achieved with the λ-red mediated homologous recombination technique^36^. We employed two compatible vectors for controlling the expression of proteins: pTrc99A encodes resistance to ampicillin and pBAD34 encodes resistance to chloramphenicol. Strains, plasmids, and primers used in this work are listed in the Supplementary Tables 2–4.

### Cell culture

We grew overnight cultures in TB (tryptone broth) at 30 °C from colonies freshly streaked on solid media plates (LB agar), supplemented with antibiotics when appropriate (100 μg/mL ampicillin, 25 μg/mL chloramphenicol). We grew day cultures by diluting overnight cultures in fresh 10 mL TB at 33 °C in a shaking incubator set at 175–200 RPM. The cultures were supplemented with ampicillin (100 μg/mL) and chloramphenicol (25 μg/mL), as appropriate. To induce expression from the *ptrc99A-cheY-eyfp* plasmid, we added 15 μM isopropyl-β-d-thiogalactoside (IPTG) to the day cultures. To vary the expression from the *pBAD34-motA-motB* plasmid, we added 1 × 10^−5^ to 6 × 10^−5^% l-arabinose to the day culture.

### Tethered cell assays

Upon reaching an OD_600_ ~ 0.5–0.6, the day culture was washed twice with motility buffer (MB: 0.01 M potassium phosphate, 0.067 M NaCl, 0.1 mM EDTA, 1 μM methionine, 10 mM lactic acid). The cell pellet obtained from the wash cycles was resuspended in 1 mL MB. We sheared the flagellar filaments by rapidly pushing the cell suspension through a 10 cm polyethylene tubing (0.58 mm inner diameter) ~50–75 times with the aid of two syringes with 21 gauge adapters. The sheared suspension was again washed in MB and resuspended in 400 μL MB. The concentrated cell suspension was layered on top of a 12 mm diameter coverslip (Fisher Scientific). The cells were allowed to settle and tether to the coverslip surface for 5–7 min. The coverslip was then used to seal a perfusion chamber with the side with adhering cells facing inward^37^. The outlet of the perfusion chamber was connected with a 0.58 mm ID mm tubing to a syringe pump (Chemyx Fusion 200) that withdrew fluid at ~120 μL/min. The inlet of the perfusion chamber was connected to a reservoir containing MB. Continuous perfusion with fresh MB eliminated oxygen gradients and removed cells that did not adhere to the surface.

### Bead assays

We treated the 12 mm coverslips with 0.01% poly-l-lysine. We then rinsed off unbound poly-l-lysine multiple times with MB. Next, we layered the sheared cells onto the coverslip. We waited ~5–7 min to allow the cells adequate time to settle and adhere to the surface. We then added ~10 μL of a washed 2 μm bead suspension. We waited 5 min for the beads to sediment and tether to the flagellar stubs. Finally, the coverslip was used to seal the perfusion chamber.

### TIRF and phase measurements

We coupled a 100 mW 514 nm laser (Cobolt Fandango) into a TIRF microscope (Nikon Eclipse Ti-E) equipped with a 60× TIRF objective and aligned the laser to generate an evanescent field with a characteristic decay length of ~100 nm. We performed all the test and corresponding control experiments for each subplot in Fig. 1 in the same timeframe (consecutive days). This minimized systematic errors due to the gradual drift in TIRF alignment that occurs over extended time-periods. Tethered cells were illuminated with the laser, and the emissions were filtered and relayed to a back-illuminated, cooled (−60 °C), EMCCD camera (Andor iXon Ultra). A clean-up bandpass (555/55, Chroma Inc.) ensured that only the TIRF emissions entered the EMCCD camera. This setup enabled us to visualize the cells in the phase contrast channel simultaneously. Phase illumination was achieved with halogen light filtered with a bandpass filter (745/90, Chroma Inc.). A dichroic mirror (680 nm cut-off, Chroma Inc) split the TIRF emissions and the phase signals. Before obtaining the fluorescent emissions, we brought the tethered cells into focus in the phase channel. Our microscope is fitted with a Perfect Focus System (PFS, Nikon Inc), which helps maintain focus over long periods. We then exposed the cells to the laser while recording three consecutive images on the EMCCD camera, using an exposure time = 80 ms.

The rotation of tethered cells and large (2 μm) beads was recorded with phase contrast microscopy at 20–100 frames/s with an IDS-uEye monochrome camera UI-3240LE-M-GL. A faster camera (Fastec, Il5-L-M SVGA) was used to record the rotation of 300 nm beads at 1500 frames/s under dark-field illumination.

### Optical traps

We formed an optical trap with an IR laser (976 nm wavelength, ALS-IR-976-10-I-SF, Azurlight). First, we expanded the beam to ~10–12 mm diameter with a pair of lenses (Thorlabs Inc) that formed a Galilean telescope. The expanded beam was subsequently relayed with the help of another pair of lenses into the TIRF arm of a Nikon Ti-E microscope. A pair of dichroic mirrors reflected the beam to overfill the back-aperture of a 60× TIRF objective (Nikon Inc). See Supplementary Fig. 2 for details.

### Stalling of tethered cells

We treated 4.5 μm polystyrene beads (Polysciences, Inc.) with a 0.01% poly-l-lysine solution before introducing the beads into the perfusion chamber. The optical trap was used to trap a single bead at a time. Rather than moving the trap, we translated the microscope stage with a joystick (MS2000-XY, Applied Scientific Instrumentation Inc), which allowed us to precisely position the bead into the path of a rotating cell.

In an alternate version of this experiment, we used a micromanipulator (Eppendorf TransferMan^®^ 4r) to push beads that were settled on the bottom coverslip of the chamber with microcapillaries (Piezo Drill Tip ICSI, 25° tip angle, 6 μm inner diameter, 6 mm flange).

### Image analysis

To analyze bead rotation, we employed particle-tracking approaches that accurately detect the centroid of the bead in each image frame^9^. The bead positions followed circular tracks, which helped calculate the speed and the direction of rotation. The tethered cells were analyzed with cell-tracking algorithms developed previously^9^, which fit ellipses to binarized images of the cell. Each fit provided the orientation of the cell in that image. The speed and the direction of rotation were determined from the changes in the orientation with time. To calculate the point of tether, we plotted the center-of-mass of each fitted ellipse over the entire time-series data. As can be seen in Supplementary Fig. 3, the center-of-mass followed a circular track. The center of the circular track was determined by fitting a circle, which coincided with the point of tether (and the location of the motor). The point of tether enabled us to determine the motor location in the corresponding TIRF channel.

In the case of tethered cells lacking stator proteins, we recorded movies for a long duration to allow the cells to rotationally diffuse along the point of tether. The point of tether could be readily determined if the rotation was ~180° or more. For cells that did not undergo at least a 180° turn, we used hydrodynamic flows to force rotation. Turning the flow in the perfusion chamber on and off was adequate to cause the cell to rotate and align with the flows. This method allowed us to determine the point of tether.

The TIRF images were analyzed following our previous algorithms^9^. Because photobleaching destroys the fluorescence signal, we only used the first of the three consecutive TIRF images to determine the intensity of CheY-EYFP localized at the tethered motor. First, we used a spatial bandpass filter to filter out background noise. The algorithm then detected an intensity peak that coincided with the motor, around which we placed a 350 nm digital mask. We then summed up the intensities of the pixels within the mask to quantitatively determine the intensity of the localized fluorescent puncta.

### Statistics and reproducibility

Cells were randomly sampled from large populations of cells; each motor was obtained from an independent cell. Two-tailed Student’s *t*-tests were used to compare different populations of motors without any corrections. Differences between data obtained from different cultures of the same strain were not statistically significant.

### Reporting summary

Further information on research design is available in the Nature Research Reporting Summary linked to this article.

## Supplementary information

Supplementary Information

Description of Additional Supplementary Files

Supplementary Movie 1

Reporting summary

## Data Availability

All data are included in the manuscript and supplementary text. Source data are provided with this paper.

## Source data

Source Data

## Materials availability

Strains and plasmids are available from corresponding author on reasonable request. Source data are provided with this paper.

## Code availability

No new algorithms or codes were developed in this work other than minor modifications to previously-published MATLAB routines^9^. Codes are available from corresponding author on reasonable request.
