# Supplementary material for: Mechanosensitive recruitment of stator units promotes binding of the response regulator CheY-P to the flagellar motor
Source: Nat Commun. 2021 Sep 14;12:5442. doi: 10.1038/s41467-021-25774-2 (PMC8440544; doi:10.1038/s41467-021-25774-2)
Supplement: Supplementary file 1 — Supplementary Information [file 41467_2021_25774_MOESM1_ESM.pdf]

Mechanosensitive recruitment of stator units promotes binding of the response regulator CheY-P to the flagellar motor

Jyot D. Antani[1,2,3], Rachit Gupta[1], Annie H. Lee[1], Kathy Y. Rhee[1], Michael D. Manson[4], and Pushkar P. Lele[1,*]

[1] Artie McFerrin Department of Chemical Engineering, Texas A&M University, College Station, TX (USA) – 77843-3122.

[2] Present address: Department of Ecology & Evolutionary Biology, Yale University, New Haven, CT (USA) 06520-8106.

[3] Present address: Department of Molecular, Cellular, and Developmental Biology, Yale University, New Haven, CT (USA) 06520-8103.

[4] Department of Biology, Texas A&M University, College Station, TX (USA) 77843-3258.

* Correspondence to: plele@tamu.edu

Supplementary Note 1: Predicting CW$_{bias}$ versus [CheY-P] relationship from CW$_{bias}$ distributions

The random variables B and C represent the variability in CW$_{bias}$ and [CheY-P] in a cell population, respectively. The random variable C (CheY-P level) is assumed to be normally distributed with a mean μ and standard deviation σ. The bias B at each C is calculated from the Hill function:

$$B = \frac{C^h}{C^h + K_D^h} \qquad \text{Equation 1}$$

where h and K_D represent the Hill coefficient and the dissociation constant, respectively. We derive the probability density for the bias from the following expression [1]:

$$P(B) = f(\phi)\frac{d\phi}{dB}$$

where,

$$\phi(B) = C = K_D\left(\frac{B}{1-B}\right)^{\frac{1}{h}} = K_D\left(\frac{1}{B}-1\right)^{-\frac{1}{h}}$$

and $f(\phi)$ is the Gaussian function that describes the [CheY-P] distribution.

The expression for the CW$_{bias}$ distribution is:

$$P(B) = \frac{K_D}{hB^2\sigma\sqrt{2\pi}}\left(\frac{1}{B}-1\right)^{-\frac{1}{h}-1}\exp\left(-\frac{1}{2}\frac{\left(K_D\left(\frac{1}{B}-1\right)^{-\frac{1}{h}}-\mu\right)^2}{\sigma^2}\right) \qquad \text{Equation 2}$$

We assumed h = 10 and K_D = 3.1 μM for motors with a full complement of stator units (high torque group) based on an earlier work [2]. This is a reasonable assumption as the measurements of Cluzel and co-workers were performed at high loads where the stator carries a full complement of stator units. We used a nonlinear least-square approach to fit equation 2 to the CW$_{bias}$ distribution in the high torque group. This yielded

$\mu = 5.3 \pm 0.5$ μM (mean ± SEM) for the *ΔcheRcheBcheZ* cells. For the medium and low torque groups, we assumed that the μ and σ remained fixed while K_D was a free parameter. The fitted values of K_D for the different groups are shown in **Supplementary Table 1**. The fits to the CW_{bias} distributions are indicated in the plots.

The nature of K_D's dependence on torque did not change when we assumed different values for μ and σ.

Supplementary Note 2: Estimation of CW_{bias} at varying viscous loads

The CW_{bias} was calculated from CCW-to-CW ($k_{CCW\to CW}$) and CW-to-CCW ($k_{CW\to CCW}$) switching rates from previous works [3,4]:

$$CW_{bias} = \frac{k_{CCW\to CW}}{k_{CCW\to CW} + k_{CW\to CCW}}$$

Supplementary Figures

Raw data for CW_{bias}

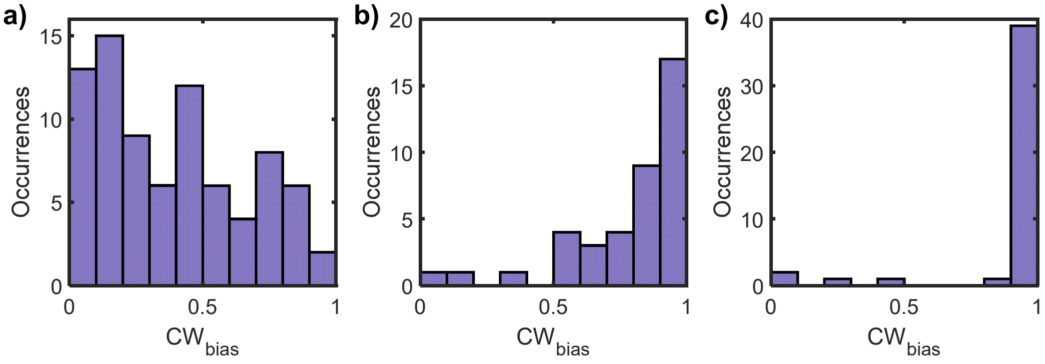

Supplementary Figure 1. Raw data for **Fig. 2B**: CW_{bias} histograms for the populations with **a)** low, **b)** medium, and **c)** high N_{st}. Source data are provided as a Source Data file.

Optical Trapping and TIRF Setup

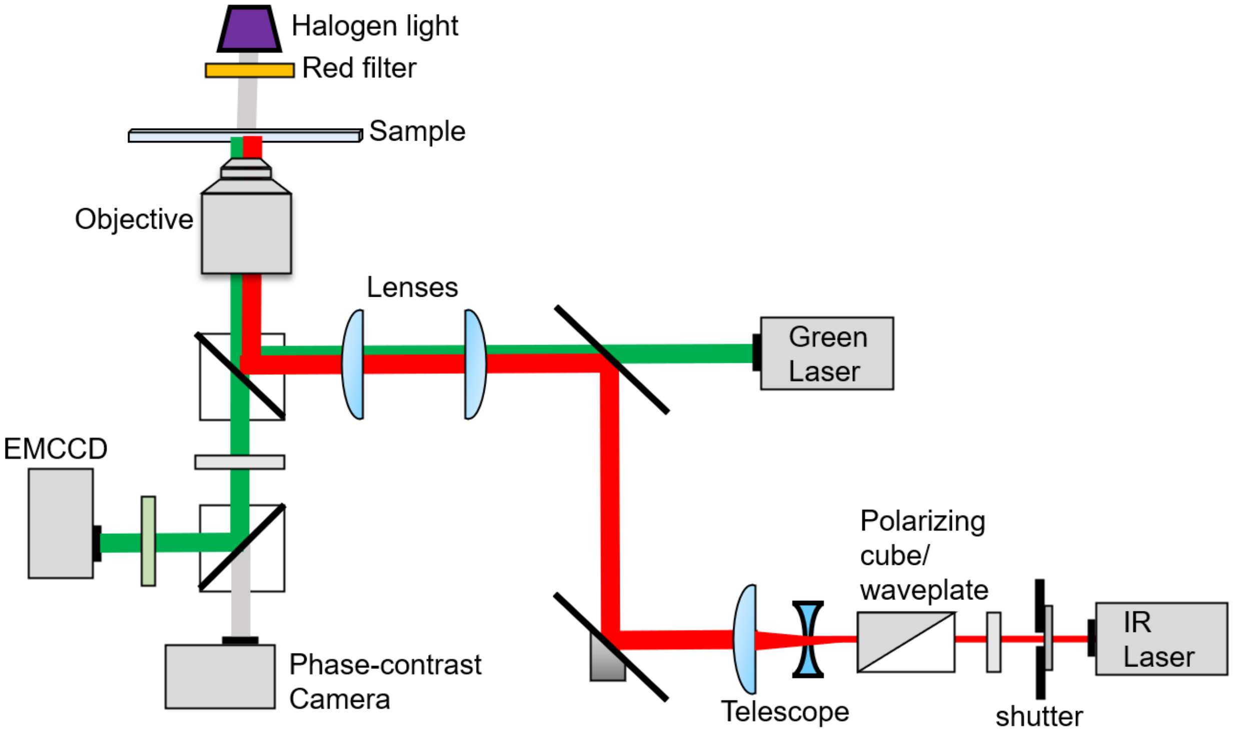

Supplementary Figure 2. Setup for combined optical trapping (red laser) and TIRF illumination (green).

Point of tether in tethered cells

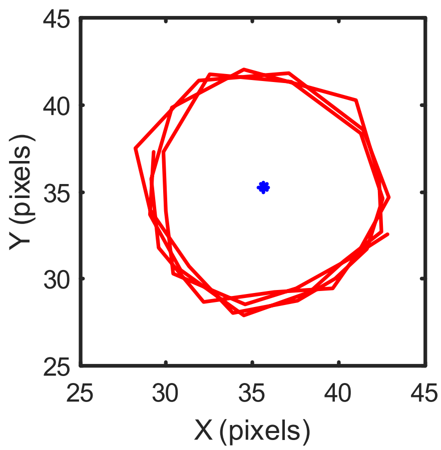

Supplementary Figure 3. The red trajectory shows the movements of the center-of-mass of a tethered cell. The center-of-mass was obtained from each image by fitting an ellipse to the cell. The tether point (motor location) coincides with the center of a circular fit to the trajectory. Source data are provided as a Source Data file.

Supplementary Tables

Supplementary Table 1: Nonlinear least-square fits

<table>
<tr><th>High N_{st}</th><th>Medium N_{st}</th><th>Low N_{st}</th></tr>
<tr>
<td>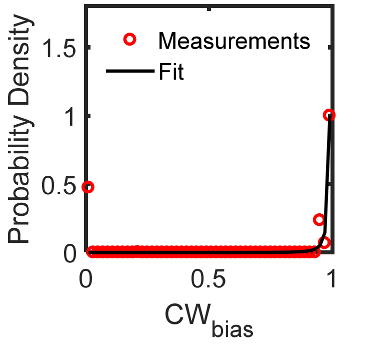
</td>
<td>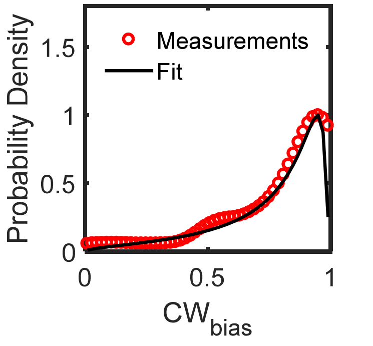
</td>
<td>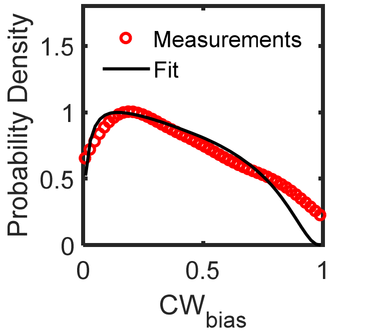
</td>
</tr>
<tr><td>$K_D = 3.1$ μM (mean ± SEM)</td><td>$K_D = 4.5 \pm 0.0$ μM (mean ± SEM)</td><td>$K_D = 5.6 \pm 0.0$ μM (mean ± SEM)</td></tr>
</table>

Supplementary Table 2: Plasmids

Plasmid	Plasmid #	Resistance	Cloning Sites
pBAD34-*motAmotB*	pPL39	Chloramphenicol	KpnI & SalI
pTrc99A-*eyfp-cheY*	pPL29	Ampicillin	KpnI & XbaI

Supplementary Table 3: Strains

Strain name	Lab Strain#	Genotype	Plasmid
MotAB+, Fig 1B	PL240	*fliCst ΔcheRcheBcheYcheZ*	pTrc99A-*eyfp-cheY*
Δ*motAB*, Fig 1B	PL239	*fliCst ΔcheRcheBcheYcheZ, ΔmotAmotB*	pTrc99A-*eyfp-cheY*
Δ*motAB*/p(empty), Fig 1C	PL349	*fliCst ΔcheRcheBcheYcheZ, ΔmotAmotB*	pTrc99A-*eyfp-cheY*, pBAD34
Δ*motAB*/p(MotAB), Fig 1C	PL350	*fliCst ΔcheRcheBcheYcheZ, ΔmotAmotB*	pTrc99A-*eyfp-cheY*, pBAD34-*motAmotB*
MotAB+, Fig 1D	PL233	*fliCst fliM-eYFP(A206K)-fliM ΔcheY*	
Δ*motAB*, Fig 1D	PL333	*fliCst fliM-eYFP(A206K)-fliM ΔmotAmotB ΔcheY*	
Δ*cheRcheBcheZ*, Fig 2A, B	PL254	*fliCst ΔcheRcheBcheZ ΔmotAmotB*	pBAD34-*motAmotB*

Δ*cheRcheB*, Fig 2E	PL138	*fliC*st Δ*cheRcheB*	

Supplementary Table 4: Primers

Primer	Sequence	Product/purpose
motAB_fwd_KpnI<br>motAB_rev_SalI	CGTGGTACCGTCAACAGTGGAAGGATG<br>TTAGTCGACTCACCTCGGTTCGGC	pBAD34-*motAmotB*
EYFP_KpnI_fwd<br>cheY_rev_XbaI	GGTACCCCATGGTGAGCAAGG<br>CTCTAGAGCTCACATGCCCAGTTT	pTrc99A-*eyfp-cheY*
CheRCheBCheZ_P1<br>NewP4_UsewithOldP1	CTGATCGAAGAGTCAGTCAAT<br>GCCAAACCAGATAACACTAAC	Amplification of Δ*cheRcheBcheYcheZ*, Δ*cheRcheB*, and Δ*cheY* DNA for construction of chemotactic mutants
MotAB_seqP1<br>motAB_seqP2New	AAGCAGAAGAAGGACCACTGC<br>GCGCGAAAGGATAATTCGTCG	Amplification of Δ*motAmotB* DNA for construction of *mot* mutants

Supplementary References

1. Lele, P. P., Shrivastava, A., Roland, T. & Berg, H. C. Response thresholds in bacterial chemotaxis. *Sci. Adv*. **1**, e1500299, doi:10.1126/sciadv.1500299 (2015).
2. Cluzel, P., Surette, M. & Leibler, S. An ultrasensitive bacterial motor revealed by monitoring signaling proteins in single cells. *Science* **287**, 1652-1655, doi:8310 [pii] (2000)
3. Fahrner, K. A., Ryu, W. S. & Berg, H. C. Biomechanics: bacterial flagellar switching under load. *Nature* **423**, 938-938 (2003).
4. Yuan, J., Fahrner, K. A. & Berg, H. C. Switching of the bacterial flagellar motor near zero load. *J. Mol. Biol.* **390**, 394-400 (2009).
