# Supplementary material for: Mechanosensitive recruitment of stator units promotes binding of the response regulator CheY-P to the flagellar motor
Source: Nat Commun. 2021 Sep 14;12:5442. doi: 10.1038/s41467-021-25774-2 (PMC8440544; doi:10.1038/s41467-021-25774-2)
Supplement: Supplementary file 2 — Description of Additional Supplementary Files [file 41467_2021_25774_MOESM2_ESM.pdf]

Description of Additional Supplementary Files

File Name: Supplementary Movie 1
Description: Movie shows the stalling of a representative tethered cell with an optically trapped latex bead. The bead was translated until it lay in the path of cell rotation.
